# Supplementary material for: Modelling human neuronal catecholaminergic pigmentation in rodents recapitulates age-related neurodegenerative deficits
Source: Nat Commun. 2024 Oct 11;15:8819. doi: 10.1038/s41467-024-53168-7 (PMC11470033; doi:10.1038/s41467-024-53168-7)
Supplement: Supplementary file 1 — Supplementary Information [file 41467_2024_53168_MOESM1_ESM.pdf]

Modelling human neuronal catecholaminergic pigmentation in rodents recapitulates age-related multisystem neurodegenerative deficits

SUPPLEMENTARY FIGURES

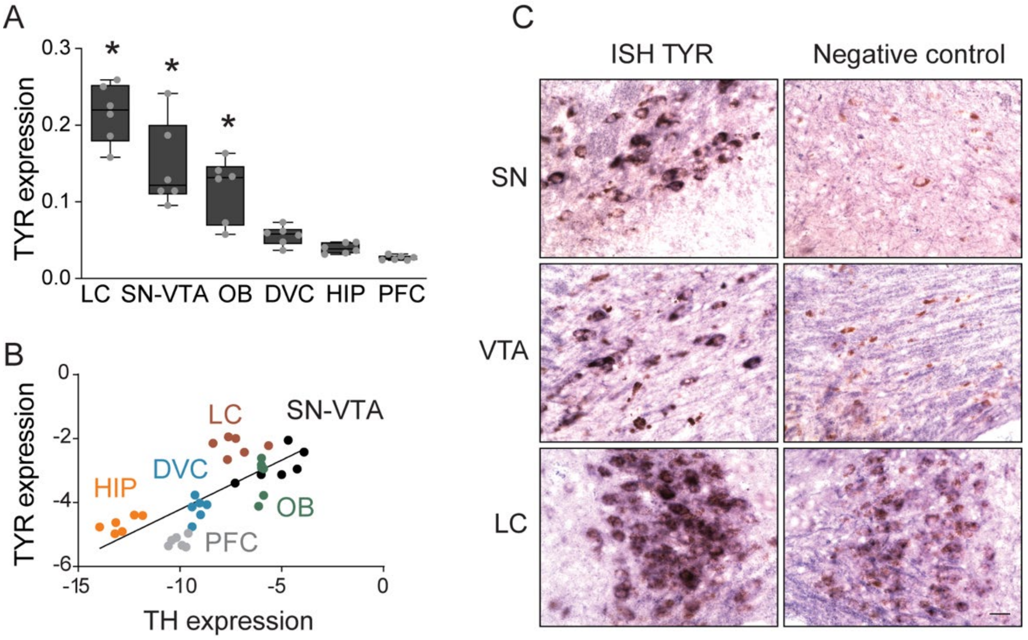

Supplementary Fig. 1. TYR expression in tgNM mice. (**A**) TYR gene expression measured in catecholaminergic (Locus Coeruleus [LC], Substantia Nigra-Ventral Tegmental Area [SN-VTA], Olfactory Bulb [OB], Dorsal Vagal complex [DVC]) and non-catecholaminergic (Hippocampus [HIP] and Prefrontal Cortex [PFC]) brain regions from adult tgNM mice. (**B**) Correlation between tyrosine hydroxylase (TH) and TYR expression levels in catecholaminergic and non-catecholaminergic brain regions from adult tgNM mice. (**C**) TYR gene expression by *in situ* hybridization (ISH) in sections from adult tgNM mice (representative image from 2 mice). Scale bar: 25 µm. In A; *$p \leq 0.05$ compared with PFC. In B; linear regression $p \leq 0.05$, R2=0.5613, y=0,3087*x-1,131. Box plots: median, min-max values and individual dots for each animal. Genotypes, ages, sample sizes and statistical analyses (Source Data). Source data are provided as a Source Data file.

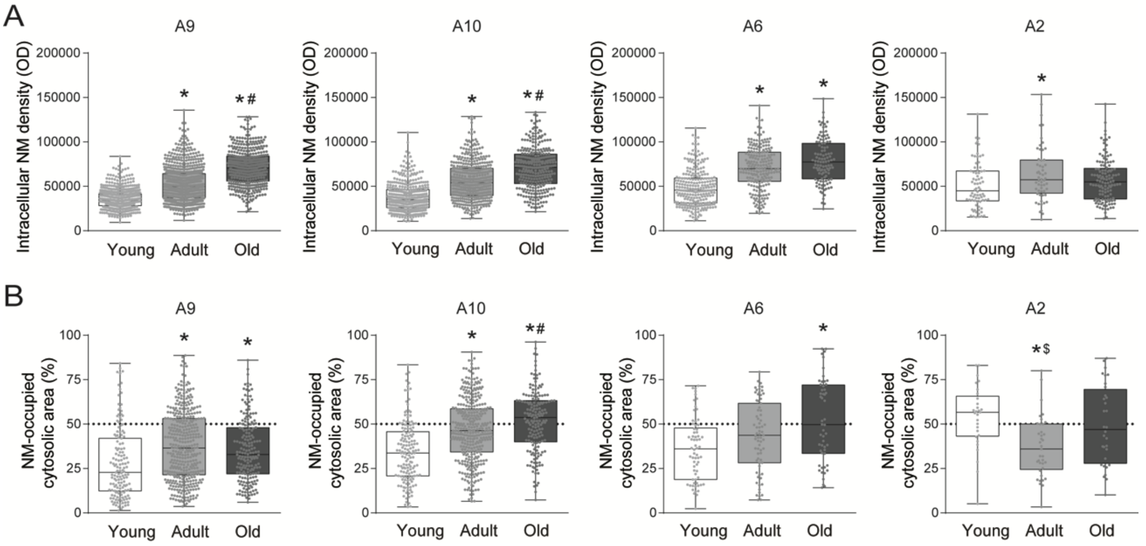

Supplementary Fig. 2. Age-dependent NM accumulation in tgNM mice. (**A**) Quantification of intracellular NM levels in SN/A9, VTA/A10, LC/A6 and DVC/A2 brain sections (same data as Figure 2B) represented for each area at different ages in tgNM mice. (**B**) Percentage of cytosolic area occupied by NM quantified in H&E-stained paraffin sections from tgNM mice. In A-B; *$p \leq 0.05$ compared with young tgNM, #$p \leq 0.05$ compared with adult tgNM, \$$p \leq 0.05$ compared with old tgNM mice. Box plots: median, min-max values and individual dots for each neuron. Genotypes, ages, sample sizes and statistical analyses (Source Data). Source data are provided as a Source Data file.

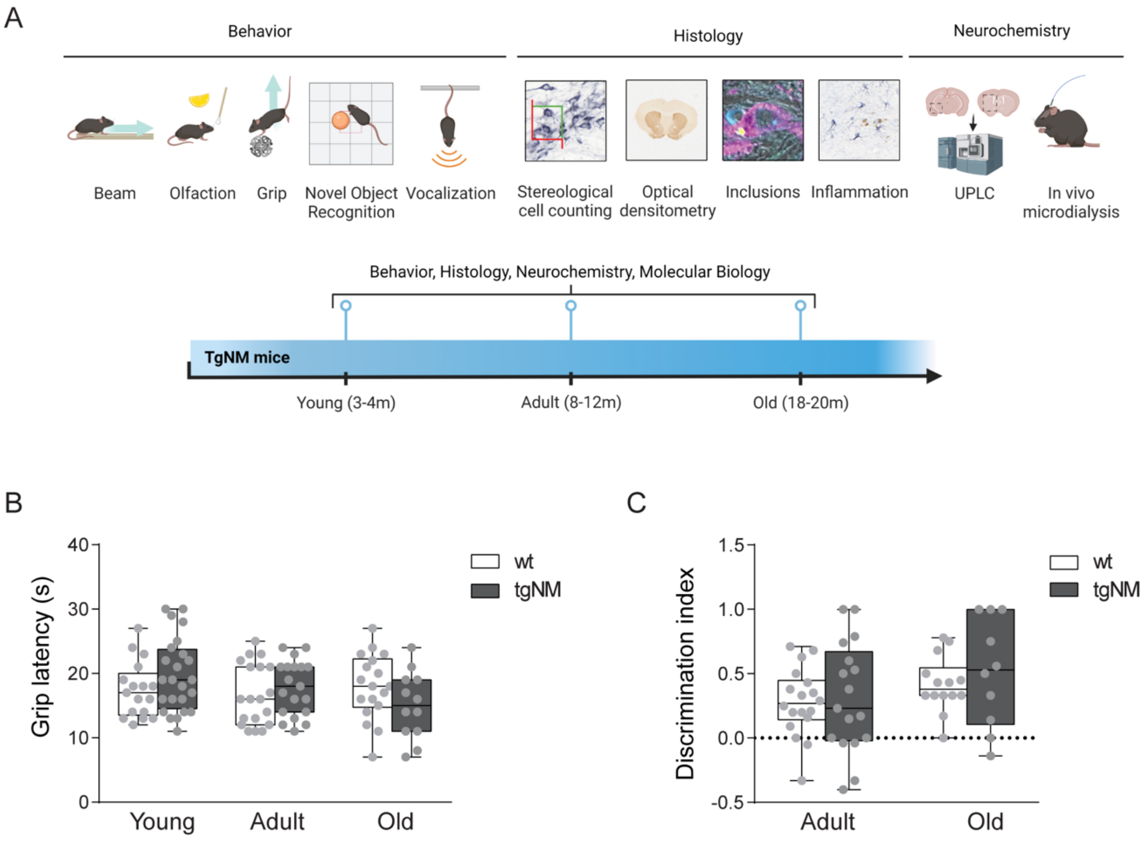

Supplementary Fig. 3. Dopaminergic dysfunction and behavioral tests in tgNM mice. (A) Experimental design for the neuropathological and functional characterization of the dopaminergic function in tgNM and wt mice. Created in BioRender. Nicolau, A. (2022) BioRender.com/j49t012. (**B**) Quantification of grip latency time (s). (**C**) Discrimination index in the novel object recognition test. Box plots: median, min-max values and individual dots for each animal. Genotypes, ages, sample sizes and statistical analyses (Source Data). Source data are provided as a Source Data file.

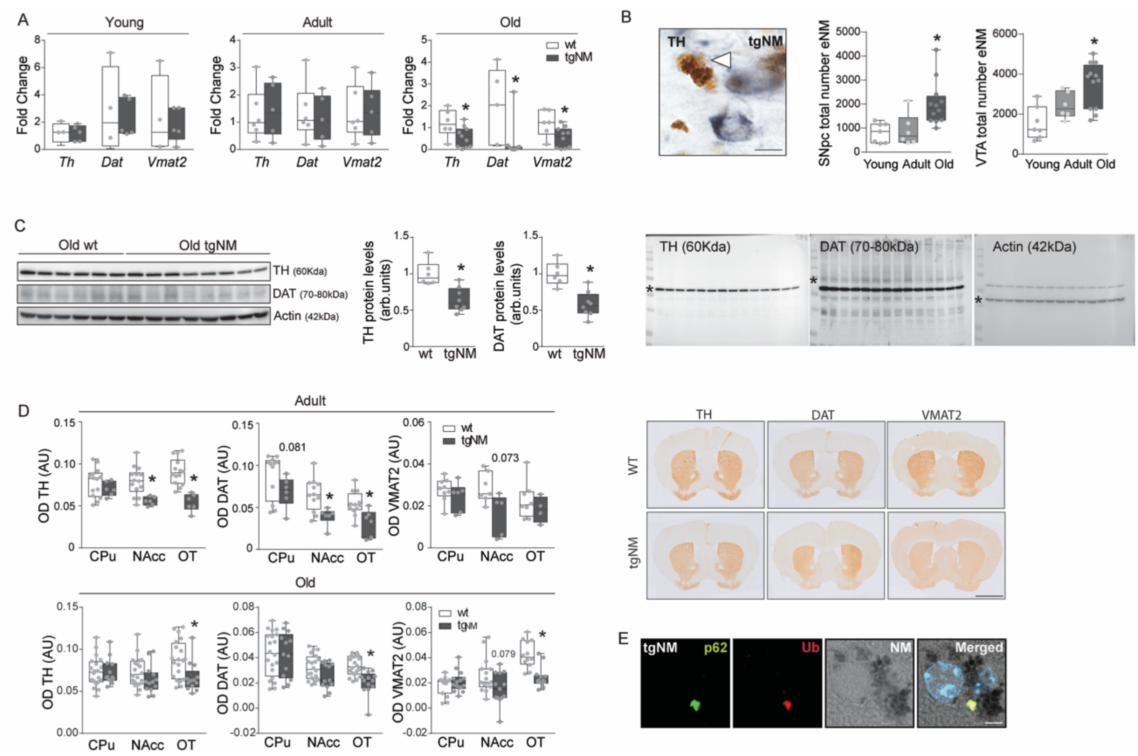

Supplementary Fig. 4. Dopaminergic dysfunction and PD-like neuropathology in tgNM mice. (**A**) Expression of DA markers in SN-VTA homogenates. (**B**) *Left*, TH-immunostained SNpc section showing eNM (white arrowhead). TH, blue; unstained NM, brown. Scale bar: 20 µm. *Right*, number of eNM granules in SNpc and VTA sections in tgNM mice. (**C**) Striatal protein levels of DA markers TH and DAT in old tgNM and wt mice. (Top left, representative images; Top Right, quantification; Bottom, uncropped versions of the three blots). (**D**) Density of DA fibers in striatum (CPu), nucleus accumbens (NAcc) and olfactory tubercle (OT) of TH, DAT, VMAT2 immunostaining in adult and old tgNM and wt mice. (E) SNpc and VTA sections exhibiting NM-laden neurons with cytoplasmic inclusions immunopositive for p62, green and Ubiquitin (Ub), red; Hoechst (blue); NM, dark grey. Scale bar: 5 µm. In A, C, D; $*p \leq 0.05$ compared with wt littermates. In B; $*p \leq 0.05$ compared with young and adult tgNM (SN) and to young tgNM (VTA). Box plots: median, min-max values and individual dots for each animal. Genotypes, ages, sample sizes and statistical analyses (Source Data). Source data are provided as a Source Data file.

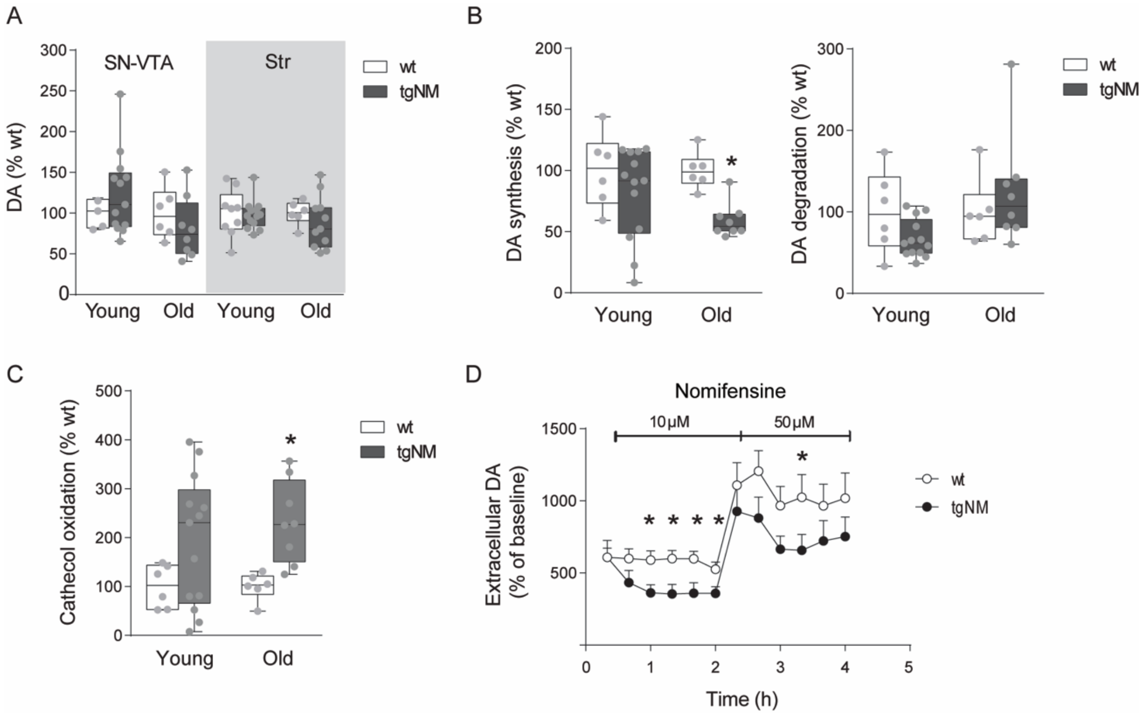

Supplementary Fig. 5. Altered dopaminergic metabolism and neurotransmission in tgNM mice. (**A-C**) Striatal and SN-VTA DA levels and SN-VTA DA synthesis, DA degradation and catechol oxidation in tgNM and wt mice. **(D)** Striatal DA release. In B, C; $*p \leq 0.05$ compared with wt littermates. Box plots: median, min-max values and individual dots for each animal. In D; $*p \leq 0.05$ compared with wt littermates. Points in the graph represent mean±SEM at each time point indicated. Genotypes, ages, sample sizes and statistical analyses (Source Data). Source data are provided as a Source Data file.

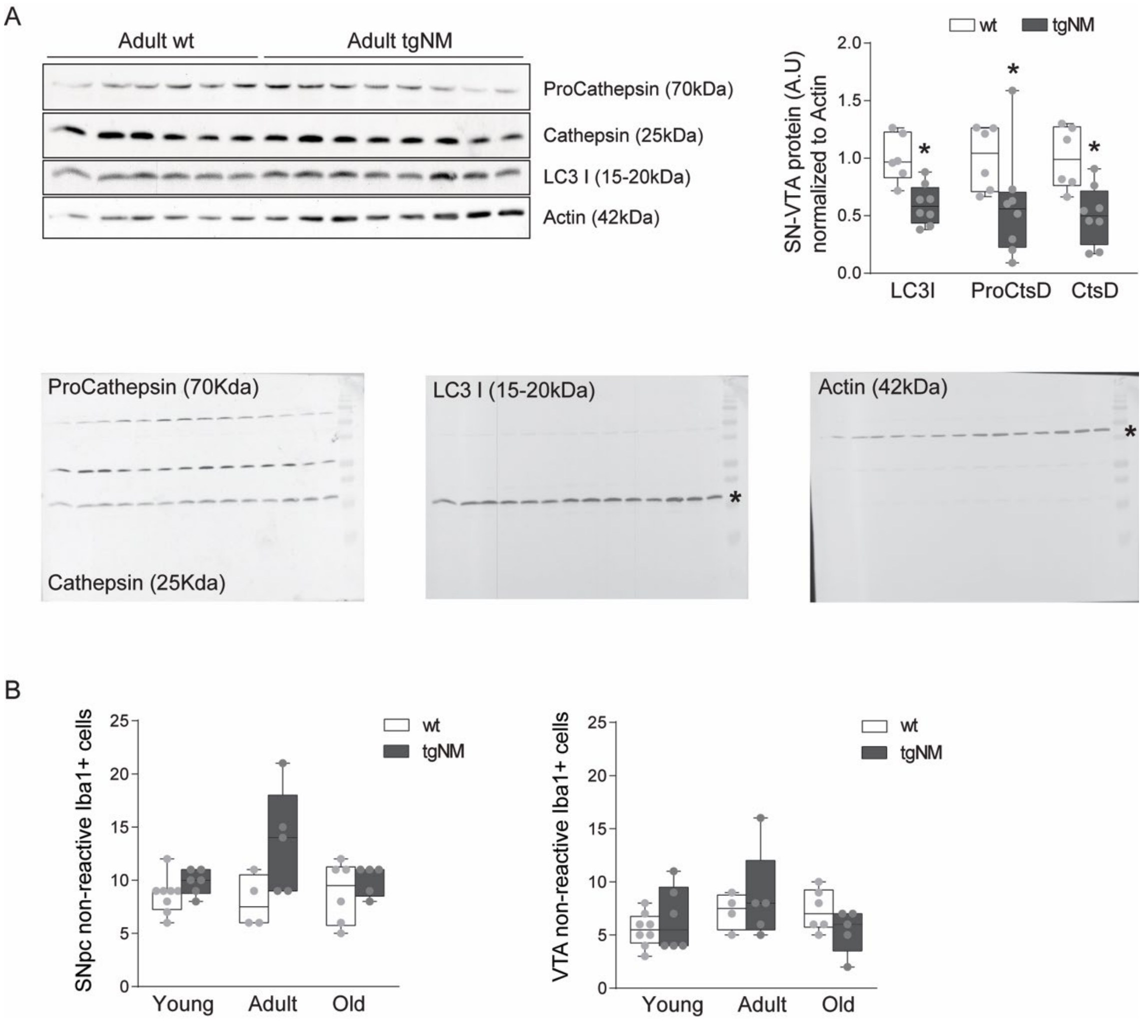

Supplementary Fig. 6. Altered autophagic-lysosomal and immune function in tgNM mice. (**A**) Top *left*, representative images of SN-VTA protein levels of autophagic markers (pro-cathepsin and cathepsin, LC3I) in adult tgNM and wt mice homogenates; Top *right*, quantification of the protein levels of autophagic markers relative to Actin levels in adult SN-VTA tgNM and wt mice homogenates. *Bottom*, uncropped versions of the blots. (B) Number of Iba-1-positive non-reactive microglia in SNpc and VTA sections from tgNM and wt mice. In A; *$p \leq 0.05$ compared with wt littermates. Box plots: median, min-max values and individual dots for each animal. Genotypes, ages, sample sizes and statistical analyses (Source Data). Source data are provided as a Source Data file.

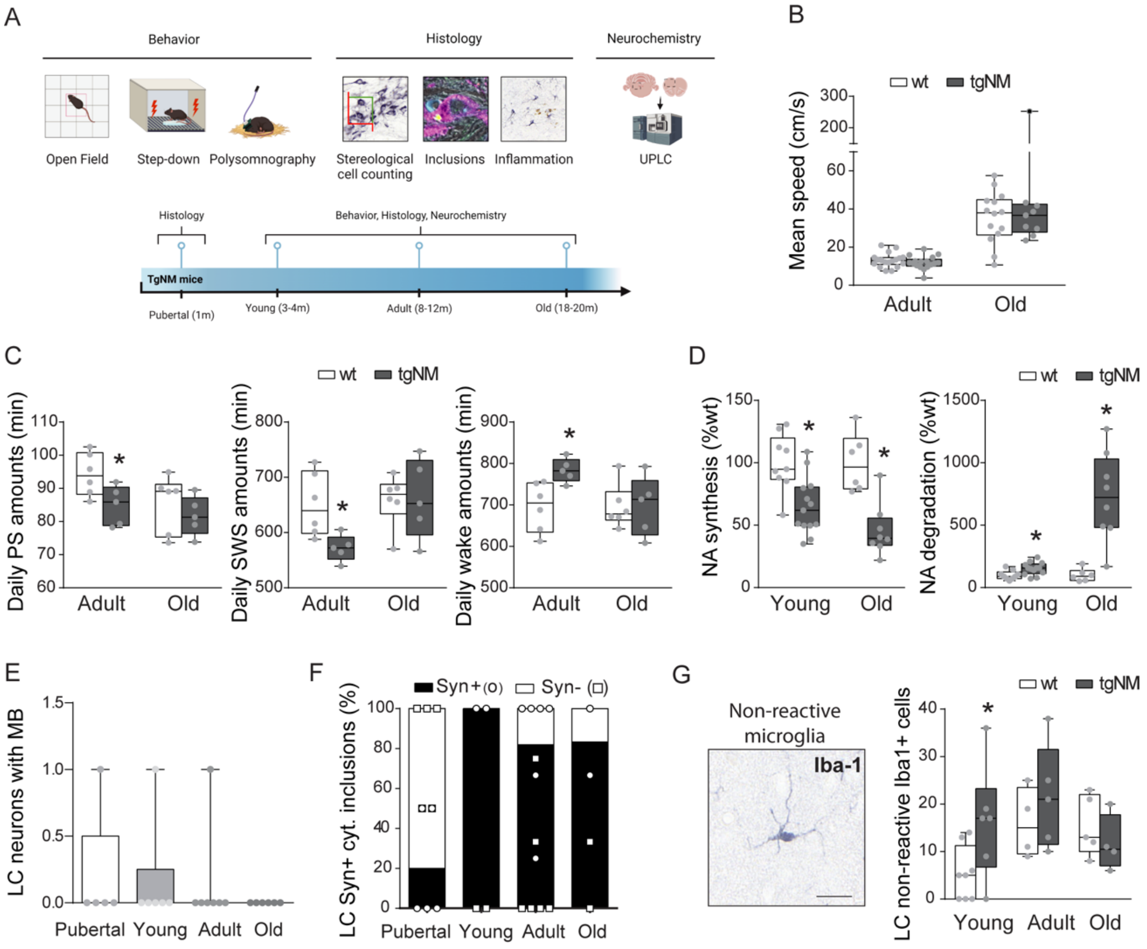

Supplementary Fig. 7. Noradrenergic neurodegeneration and PD-like neuropathology in tgNM mice. (**A**) Experimental design for the neuropathological and functional characterization of the noradrenergic function in tgNM and wt mice. Created in BioRender. Nicolau, A. (2022) BioRender.com/o69b980. (**B**) Mean speed of tgNM and wt mice in the open field test. (**C**) Polysomnography; amounts of distinct vigilance states (minutes per day; Paradoxical Sleep [PS], Slow Wave Sleep [SWS] and Wake) in tgNM and wt mice. (**D**) LC NA synthesis and degradation in tgNM and wt mice. (**E**) Number of LC NM-laden neurons with p-62 positive nuclear inclusions (i.e. Marinesco bodies, MB) in tgNM mice. (**F**) Percentage of LC cytoplasmic inclusions positive or negative for alpha-synuclein (Syn) in tgNM mice. (**G**) Image and quantification of Iba-1-positive non-reactive microglia in tgNM and wt mice. In C-D, G; *p≤0.05 compared with wt littermates. Box plots: median, min-max values and individual dots for each animal. Genotypes, ages, sample sizes and statistical analyses (Source Data). Source data are provided as a Source Data file.

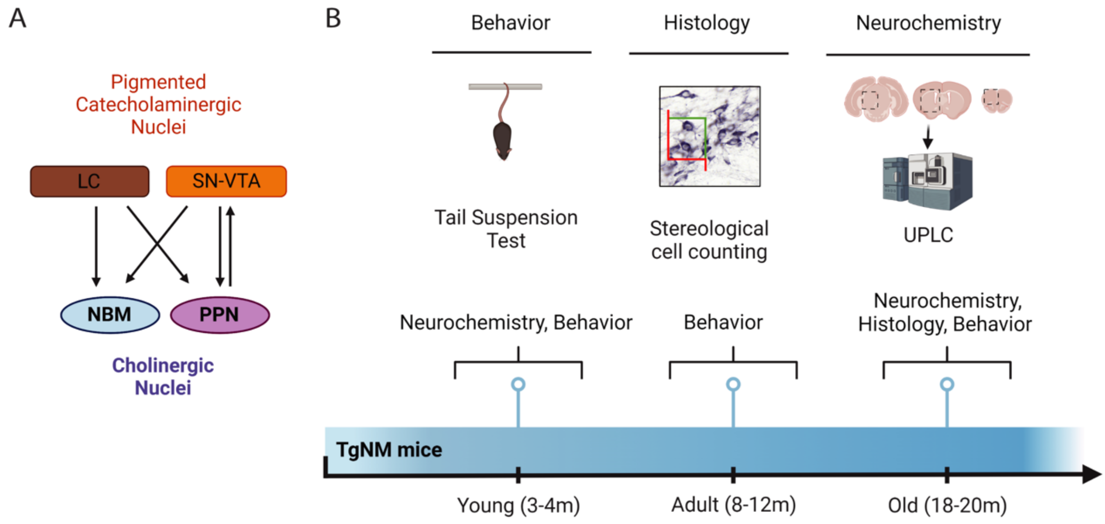

Supplementary Fig. 8. Cholinergic and serotonergic alterations in tgNM mice. (**A**) Schematic representation of nucleus basalis of Meynert (NBM) and pedunculopontine nucleus (PPN) efferent and afferent projections[1–4]. (**B**) Experimental design for cholinergic and serotonergic characterization in tgNM and wt mice. Created in BioRender. Nicolau, A. (2022) BioRender.com/a77a225. Source data are provided as a Source Data file.

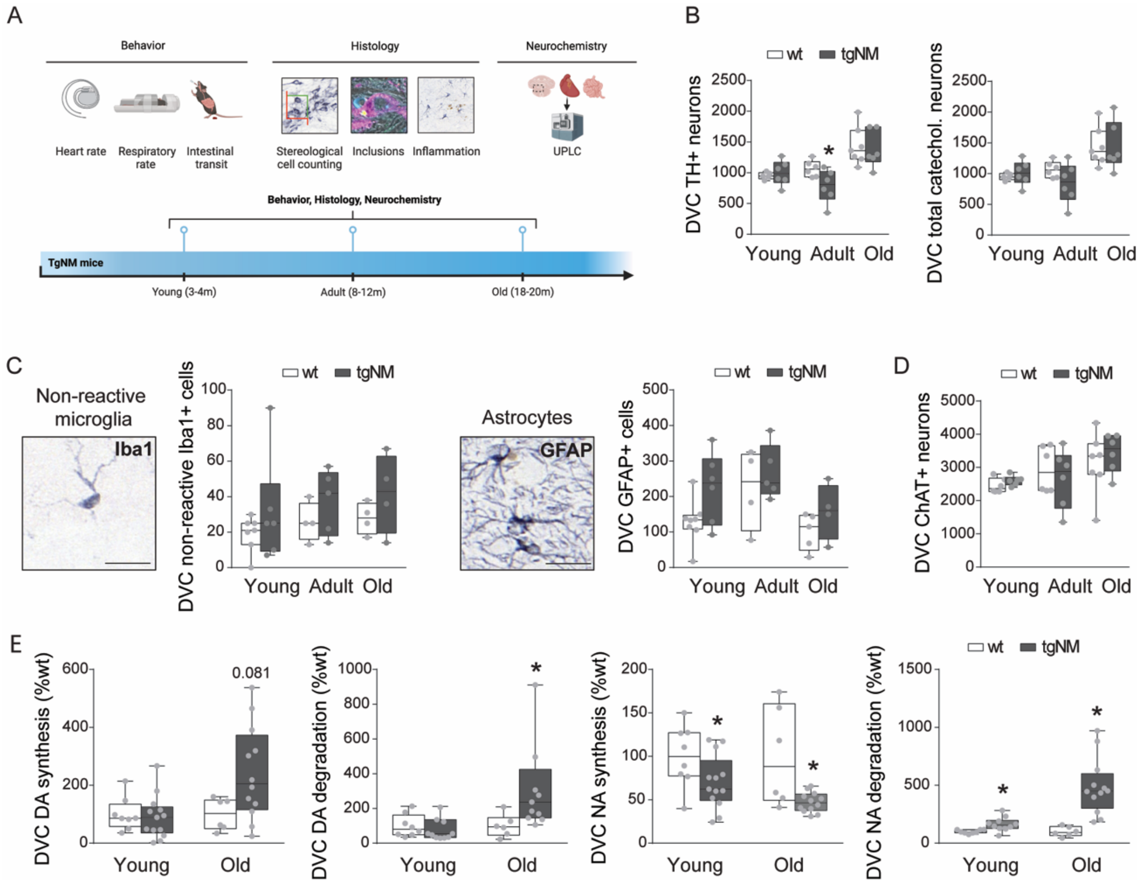

Supplementary Fig. 9. Medullary catecholaminergic nuclei neurodegeneration in TgNM mice. (**A**) Experimental design for medullary nuclei characterization in TgNM and wt mice. Created in BioRender. Nicolau, A. (2022) BioRender.com/t85c916. **(B)** Cell counts of TH-positive and total catecholaminergic cells in sections of the dorsal vagal complex (DVC) in tgNM and wt littermates. (**C**) Images and quantification of Iba1-positive non-reactive microglia and GFAP-positive astrocytes in tgNM and wt mice. (**D**) Cell counts of DVC ChAT-positive neurons in tgNM and wt mice. (**E**) Catecholamine synthesis and degradation in the dorsal medulla of tgNM and wt mice. In B, E; *p≤0.05 compared with wt littermates. Box plots: median, min-max values and individual dots for each animal. Genotypes, ages, sample sizes and statistical analyses (Source Data). Source data are provided as a Source Data file.

Supplementary Fig. 10. Plasmid map and sequence for rTHp_pcDNA4_TYR. DNA sequence used to generate tgNM mice with the human tyrosinase complementary DNA (cDNA) fused to the rat tyrosine hydroxylase promoter.

VECTOR: rTHp_pcDNA4_TYR
15873 bp

Construct map:

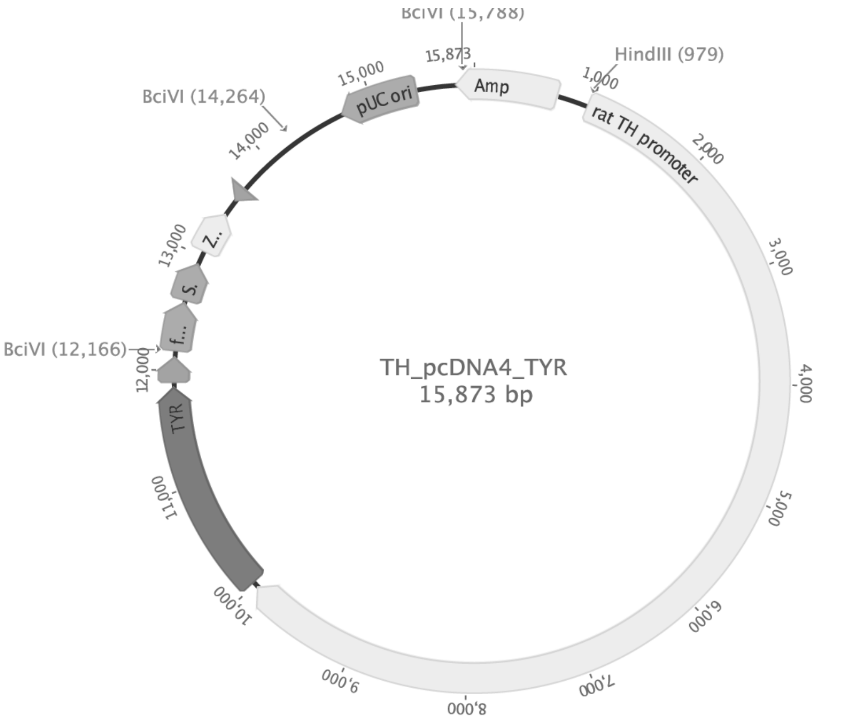

Microinjection DNA fragment:

RESTRICTION: HindIII / EcoRI / BciVI (BfuI)

Microinjection DNA fragment size: 11187 bp

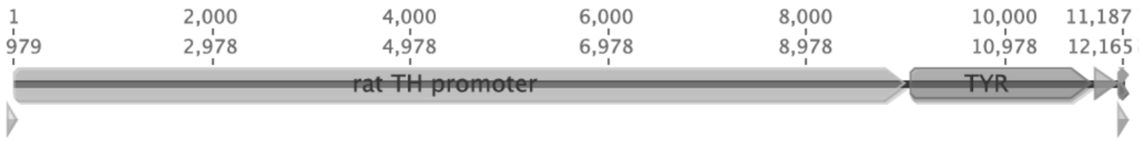

rat TH promoter
TYR gene
BGHpA site

AGCTTGGCAAGAGCTGATTTTAAAAGGTCCTGGAGAACACAGGCCAGCTCCAGTGTCAGGCGCCCTCATT
CCAACTCCAAAGTAATCGCCTGGCAAACAAGCACTGTCTAAATATCACAGAGTGTGGGTCAGGCGCACCG
CAGCCTGGAAATGTCAGTGGCGGGGGCCTCCTCCCAGCTCCAGGATTAGCCCAGGCACCTGAGCCACATG
CATTTGCCTTCAGCCTGGCTCTAGCCCCAAGAACCCGAAGAGGCCTCAGGTGTGTTCTCACTGCCAGATC
GTGGGATTGGGTCCCTGGGGAAAGGTGTTGCCCGTGGGGTCAGACTGAGCCATAAGCGGCTGTAGCCTTT
GGAATGACATACTTCCAAGTTCTCACTAGGAACCCGGAGTAGGGGCAGCCCCGTGGAGAAAGCCTGGCTG

TCCCGGGCCCTCACTGCGAGATTACTCACCCTTGTGTCCTCCCTTGTTCTCTCACTTCTACAAAACCTGA
CCACTTGCTCAAATGGCAGTGAGAAGCTGCTGGGTGGGAACTGTTGCTACCATCTGCATGGGCTCTGGGG
ATGAGGTGGCCACAGCAGCAGCTGTCATGCTTGTTAGACACAAGGTTCCTTCACCCCATAATGGGGCTGG
TGACATTGTCCTAAACAGAAGGGCTTTGCCAAGTAGAAGGATTATTGCTGGGTAAATAAGAGCCCTAAAG
GTGGAGTTTTTGTCTTCCAGGAACCTTCTTTAAATCACTAGGAACTGCCCTTAGGTCAACTGTCTCGGAT
AAGTGCATCTAGTTTTAGATCAGTCCAAACAGCCTCTATCAGTTCAAAGACCCCTACATAAAGTCTGTTG
CTCTTCCACCATGCCCCCTCCACCTCTGAGATAGCCTTGGCAGTTTAATCTCCGATAAACCTTTCTTTCA
ACTCACGGCATGTTCGTGTTTGGTGGGATCACTGTGGCCTCACAGTAGAGGATGTCCAGAGCTGTGTTTG
GCTCTGTCCCTGGACGTTCCTCTCCCTTGTATTCGTTTTGTTTTGTTTTGTTTTGTTTTTTGTTTTTAAT
CAACCCAACACAAACTAGAGTCTTCTTGAAAGAGGGAACCTTAATTGATAAAATGCCATCTCCAGGCCTA
GGAAGATGACTCAACAGTTAAGAGCACTGGCCACTTTTCCACAGGTCTTGGGTTTGATTCCCAGTCCCCA
CATGGTGGCTCACAACCAGTTCTAGTGGATCCAACTCCCTCTTCTGGCTTCTGCGGACACCAGACATACA
ATTGGTATGTGGACACATGTAGGCAAGACATTCACATACATTGAAAACAAAAAGCCTGTTGAGAGAAAAT
ATCTCCATCAGATTTACCTAGAAGCAAATCTGTATGCATTTTCTTGCTTAATGGTTGGTATGAGAGGGCC
CAGCCCACTGTGGGTGGTAACCATAGGCTGGTGATCCTGGGGTGCATAGAAAGGCAAGTTGGGAGAGGCA
CTTTGCAATGTCCCTTCATAGTCTCAGCTTAAGTCCCTGCCTCCAGGCTCCCACCCTGACTTTTCCCAGT
GATGGACAGTGACCAGGACATGTAAGCCAAATAAACTCTCTCCTTCCCAAGTGACTTTTGGTCCTGGTGT
TTATTACAGCAACAGAAATCAAGCTAGGACACTGCTTTTCTTTTTACTTCATATACTTCGATCATGTGTG
TTTGTATGTGATTAGCAGTATGGAAGCCAGAGGACAGACTAGAATATTGGTTCTTTGATGCATTGGGAGA
ATTTCCTTCCTGGGGGCTAGGAGGAGCATGGAGGAGCAGAGACTGGACAACACTTTGGCCTACCAATTGT
AGCCCTTCCCTAAGACATCAAGGCCAACTGCAATCAGGGCAGACCATCATACAGCATGTGGGGAGGAAGG
ATTGAGTACTTCAATGAGGGTCCCATGACTCTATCCCCTCCATCCATGCTGGGCGGTCATCAGTCAACAA
GCCAAGCTGAGAAATCCTCTGCAGGCTTAACTCAAGCGAAGTCATGAAGTCAAGATATTGGTTGTTTGGC
TTGGAGAATAGTGCTATGTAACAGCCGAGTCCACCTTGCCTTGAGACCATATCCCACACTGGCCTGGAGC
TCGCCAGGTAGGCTACACTGCTTTTAGCTGAGTCCTAGGGATCCTCCTGCCTCTGCCTCCTCACTGCTGG
GATTAGAAACATGCACCACCACACCTGTCTACATGTCTTCTAGGAATCAGACTAGGCCCATATGCTGTGT
AAAAAGAACTCTGTGGACTGAGTTACCTCTGTACACTACAGAGCAAGTATTTAACTGGGGGCTGGCTTAC
AATTTCAGAGGCTTAGTCCATTACAATAAGGGCAGGGAGCATAGCAGTGTGCAGAATCTGAGAGCTACAT
CCTGATCTGGAGAGAGAAAGAGAGAGAGAGAGAGAGAGAGAGAGAGAGAGAGAGAGAGAGAGAGAGACAG
ACAGACAGACACTGAACCTGGCATGGACTTTTAAAACCCCAATAACATGCCCACTCCAGCCAGGCCACAC
CCACTCTGACAAGGCCACAGTTCCTAACACTTCTCAAATAGTGCCACTCCTCAGTGACTACGCATTCAAA
CACAGCAGCCACACTAGGTAACTATGAGGCCGCAAAGCCACGGGATGGGAGCCCTCTCCACCTGGAGAAG
CCTACCCTAATGACAGAGGTGATAGGGTTATTTCTCCTGAGTTCCCCAGACTGGACCCCAAACACATTAA
AGTCTGCTCTAAACCAAGCCAAGCTGCCCCTGGGCCTCCCCTGCCAATGTCCTCCACACATGCAGAAGCT
ACACCTCCTTTGGACATCTCTTCAGTTCAGGGGGACAAAGCTCCAGAGTCTGCTGCTGTCCCATCTCCTG
TCCCTGCCCATAGGCATCCTCCATAGGCAGGGCCCTAGAAGTTTTTCTACTGTGGCTTGGCACATGTGAG
GCAAGTGAGACTGTAAGACTATCCCTGCTAAATGCTGGGGCCACTGTAACTCTGCTAACACAGCCCCGGG
CCATGCTGTGGGGAGCAGAAGCCACCTTGTAGATGAGAAGAGAGGACTATACACCACCACAAACTGCAAG
AAAGAAAGCACTCCCATTCGGAGCAGGAACCACTGCCCCTCCCTACCACAGGATGGGAGGGGGACCACCA
CCCACAGGCTCCAGTGCTTCCGTGGGCCTGGGGTGGGTAGGCAGCCGTCTTGCAATTAGACATGATTGCT
GTCACATCACCACCGTGGTCCTTGTGAGTGACTAATGGGAACTGAATGTCACTCTTATTGCTTTTACTGG
CTAATTTGTCAGACTGTCAAGTACTCTGGGAGGTGGCACGGAGGGTATTACGTGACTCACTGGTGCACAG
GTTGGGAGAGGCAAGGGCCTTAGGGGGGACAGAGGGAAGCAGGAATACCAGAGAGGACCAGGGGTCTGAC
CCATGACATTTTCGGAAAGTCTGAGGGGCATGGGAGCTGAGGTTACCCCACCTGTCACATATCGATGGCT
CTGAGTTCCTCTAGGCCCTTCTGTGTACACACATGCTTATATGAAGGCTTGGGGATATAGAACACATGCC
CTGCCCTGCATGTGCATATAGACACAATGGCATGTGCATAAGGCATGTACATGTGCATGCACATGTGCAC
ATACATGTAGTGTATTGCATTCAGACCAAATGAGGTTAGATATAGATCATGGGTCCCTGGCCTGGACACT
CATGTAGCCAGCCACTAGTGAATTTGGCATGACACCTCTCTATCATCTCAGGGTTGGTGTGTAGGTTGCC
AGGTTGGTGTGGGTTGCCAGGTGACATAGGTGTAGCTGTGAGCCTCTGCTAATGTGAGGCTAGTGTTGAG
CGCTTCAGAGAAGCCTGGTTCTAATGCTGACTTCCTGTTGGCTAGGTCTAACAGAGGGACAGGCTCTCTA
ACCTGGACTCACTCACTCTCTCTCTCTCTCTCTCTCTCTCTCTCTCTCTCTCTCTCTCTCTCTGTCTC
TCTGTCTCTCTGTCTCTCTGTCTCTTGCCTTAGTTGCACTCTGGCTATTGGTCCTCCCTCCTGGTGACCA
CCCTGACCCAGGCCCCTTGGCCTTTGAGAACGCTACACACCTAACTAAAGTCATCTCCTACTAGAAGCTC
AGAGAAGGGCCAGCCATCTCTCAGCAGATGGAGAGTTCTTTAGAAGCACCAGAGCTCCTGAGAGCTGCCA
CGAGTCACTCCCAGGAATCACCGTGAAGCTAGGGAAATCAAGGCACAAGGTACGAGAGGCTGAGCCACGC
TGGGGCTCTCTTACCTGTAGACAGTGAATCTCCAGGAATAAGGCAGGCAGAGAAGCAGTGCTGGTCTTAC
TAGCCAAAGGTGGAAGTGGTTTAGGGGAGCGAGGAGGGTGGGCTGCCTGGAAGATACTCTGGACCTGAAG

GCTTGGTGGAAGTGGGGAGCCAGGGTAGAGGAAGCAGACAGGTGGGGGCTCCTTGGGGAGAGAGGGCCTA
GAGCCTAGGTTGAGACGGGGGCTGATCAGCAGCTCTTGCCTCTGGTCTGACTTTCAACTGCCCAATTATC
CCTAAGTGTCTCCTATCGACCGACCGCAGCTGGTGCTGTCCGGTACGTGTTCTGAGTCTACAGCCCCGAG
GCTGCTGCTCCATCCGATGGCCTCGTTAGGGCTAATTGCTCTGGCATTTGGGCCTGATGAGGACAAGAAT
GGCTGGACCCTACCAGATGTCAGGGAGCTTCATTCTCTTTCCAGTCAGATTGGTGAGCTGTGTCAGGGAC
AAAGGGCCCTGCGGTCTCACTGAGCATCAGGTCCCTGCTTTACAACCCATCTCAGTCTCCTGGTGGCAGG
GAAAAGCAACCTCACTCTCCCTTCTGTCTTGGGCTCACCCTGCACAGCACTTAGGCAACTGGTCATGATG
TCTTGACCAACTTAAATCCAAGAGAAGCTCCCACTGCCTTTCAGGGTATGTCAGTTGATGATTTCATGGA
CCTGCATCTCTGTGAGCAGGAACACTGACCCCAGAGCCCATCTGGTGACAGGATTCTTGAGACCCTGGCA
TCATGTGTTTCTATAGAAAGCGATTTGGGTGGACTTGTCAAAGTGGGTGGGGTATGTGTTTGCGTGTGTG
TGTGTGGGGGTCTATAGGTATAGCATGTTTGTGAATGCATGAACATATTATAATACATGTATGCCTATGC
GATTCTGTTTGTATGTGTATATCTGTGTTCGCATATGTTTGTGTGTGCATGTGGCTGCTCCTATGTATAC
TTGTGTATGTATTTGTGTGTCTGTGTCTCCATGTATAAGCACTGTGAATCTGTATATCTGTATGTGCATA
CGTTTGTGTGTCTATGTGTCTATGTATATACCATTGTGTATATACATTTGTGTGTGCCAATCTCTCTCTG
AATGTATCTGTATATGGCCATATGTGTACATGTATGTCTTTATACATGTGTGTATGCCTATGTATGTCCT
CATATATGTGTGGTCATGTGTCTCTGTGTGTACATGCTTCTGCGTGTCTATGGTAGAAGGTCATGATGCT
GGTTGAAAGTGGCCTTTGAGCCTAGGTTGTAGAGGCATGTGTTCCAAAATGCACATAGGTTTCCATAGTG
CCCAAAGCTATGGGGTAGATCTGGGGTCCTTGCTCCAAGACCAAGGATCAGGACACCCCCTAGTCCTCTG
CCTCCTTAGTTCCATGATGTCCTGGGCAGGGAGGTCCATATCAGCAGGGTGCATACAGGATAGCACAGCA
GCCTCCACACCAGCAACACTGATGCCAGGGAGTGGGTGAGCTGTCATTATGGACAATTAAGCACCGTGCT
GAGAGAGAAAGGCCCCGTGTGCTCCCCGTCTCTGCAGGCCTAATCGCTGTGGTGACAGAGGAGGGTAATT
ATCAGGACGGCTGTGAATGGCACAGGCATTACAGGGGTGTAAAGGGCTCAGGAAGGAGGGAGGGCCTGCT
ATGCCCCCAGCACGCATCTGAGCCCAGAGCAGGCAATGGCCCACTGCACTGGCCTTGCGCTTCAGATCAG
CCCCCTTTCTTAACTGCTAGGGGATGCTTCCCAATCACTCCTCTAGGCTCTGCGGCTTGGCTTCCAGCCT
GTACGCTGTCCAGAGAGCCTTCAAAGCCTCACTTCGACCAACCAGAAGCCTCTCGTCAGCCCTGCCCTGA
CCTCGTGTGCCTCTTCAAAGTGAGATTTAGCAGCTGCAGCTGGGGGTGCCTGAGCCCCACTCATGCTGTC
TTCCTTGAAGACAGAAGTGTTGGGAGCTGAGGACCTGGGCCGTATGATCCAGAGAAGTAGTGTGCTTCTG
GGTCTCAGCTCTCCCTTCTGCAGAATGGGTCTGTCTGAAATGGAAAGGCAGGTGCCCCTCTGCAGGGCCT
AATCTGAGTCGCCATGAGTGGTTAAAAGATCCAGCTTGTCTGTGGGTGAGCTTTGAGAGGAGGCAGGGAC
CTCTAGCATGGAACAGGGCTGAGTCCTGGAAAGCTGACCAAGGGCAGGCCTAAGAGGCCTCTTGGGATTC
TTCTCATCAAAAAGGGCATGGGACACAGCTAAAGCGTCCAGGGCTCCTCTGTGCCCACAGATGCCTTAGA
TCTTGGCACAATGTAGTCAGCCAGCTCCGTGTGTGTGTGTGTGTGTTTGCATGTATCTCACAGACAGTGC
ACAATGGCCTGGATGTGAACAGAGGCAAGAGTCTGGGCCAGCAGTTGTCTCCCAGGAGGGTCCAAAGACA
TCGTATTTTCAAGTTTAGGCCAGGTGCTCACTTGGGTGAGCTCAGACACAGACAAAGGTCTGGAGAGCAC
ACATTCCCCACCCCCACCCAGCTCCTATGCAAGCACCTCCAGCCGAGACAAGAAAACGAATTAAAAAGCA
ATATTTGTGTCAGCGTAAGACATTTGCCGAAAGGTTAAATCCACACTCGTGGTGCTGCACAGCAGCCCCC
TGTGCAGGATTTGTTAGGCACAGCTCCCTCCTACCCCGTGCCACCTGAGCAAATGCCAGGCTGGGTGGGC
TGGAACCAGCCTGGGCTTGCCTCACCCTGGAATCCCCAGCACCCTCCAAAGGAGGACCCTGGGAGTGGGC
ATAGACGCCCTTCAGGTGTGGGCAACAGCCCCCAGTCCTCAGGATGAAAGGCTAAGGTGCAGCCAGCTCT
GCCTTCACGGTGGGAATGTCTCTATGTGAGCCCTTTCTGGGCTGTGAAGAACGCTCTGAGAAGGGTCCTG
GGACCCTGGATAGGCCAGAGCTGTGCTGGGCATGTAGAGACAGGAGTGGGCTAAAGCAGCAAAGGCACTG
ACCAAGGAAGAGTTCAGAGAGGAGCGTGGAATATGGGGAGGGGTTCATAGTAAGAGAGAGCAGGCAGTGG
AGAGTAAATAGTCACTGAGCCGGGGTTTATGGGGTTTGTAGGAGCTTACTCAGAGAAAGTAGATGAGAGA
TGCCATGCCAGTCTGAGTATCACAGAGCCCCAGGCTCTCCTGGGAACGGAACTGTGAGGGCCAGAAGGTC
AGCAAGGGAGGTTAGGGAGAGTTCCTTTTGTACTGACTCAGCATTTATCCTGCTCCCAGGGGGCAATGGG
GGCCAGTGAGGGATGCAGAGCAAGGCAGTGATGTGGCAGGCAGTTCCTGTTGTGAAAGAGCTGGGAAGGG
AGCGGGCTGGGCCTGGTACGTACAGCAGGCCATTTCTGAGGGTCCGAGTGCTGTCTAGGAGGTGCAGTGA
GACTTCAGTGATCAGCCAGAACAGAAGCTAAGCGGGGTGGGGACTGCGAGTTCAGGCTTCTGGGTCTTGC
AAATATCCAGAATGCTAAATCCTCAGAACCCCAGGGTGGCCATTTTCAGAGTGGGTTTTGTCCTTTGGGC
ACTTGTGCAGACTCCAATATCCAGAGGGATAAGGATGGTACTCTTCAGTACCCTTAGTGAGAGGACACTT
TTCTCTGAAGGGCTTGAATGTGCCGAGCCATTACCTGAAGGAAGGAAATGACTCCAGGGACATAGGATGG
GCCCAGCACAACTCACCTGCTACAGAGAAAGGTCCCCTCCCTGGTCTCCTTAGAGATCCTGTTTCCCTGG
CTGAGGAAGCTAGGGTGGATCTTTGTGTAAGTGGGTGTGGATGCTAACTGGAAAACAAAAGGTCACTTAC
TGTTAGACCTCGGGGTACCATGGAAGAGATGATCACTGAGTGTGCCCTTACATGGGGACCAGCTGAGAAT
GGGGCTACCACTAGCTCGAGACCATGATACAGGGAATAAGTGTGCATTTGGGGGTAGGGAGTGGCTCAGA
ATACTCTTAACCAAAGCAGAGGTTTGCTCCCACAGGAAGGTGAGGTCAGAAGGCCTTAGGGAGCTGCCAG
GGGCTAGGGTTGGCACCATCTCCCAGGCTGTGTCTTTAAGGAGATGATAATCAGAGGGATAGAACCTTGC

AAAAGTGGGCCAGTCTTGGGAATACTATAGAGGAATAGCCTTCTGGAACATTCTGTGTCTCATAGGACCT
GCCTGGGGATCCAGCCCCAGTGCCAGCACATATACCGACTGGGGCAGTGAATAGATAGTACACTTTGTTA
CATGGGCTGGGGGGAACATGGCCCATGTCCTGGAGGGGACTTTATGACAGACATCCAAAAATCCAGTGAG
AGGGCTTCTAGATTTGTCTCCAAAGGTTATAGTTCTAACATGAGCCCTTAGGAAATCCAGCATGGTTCTC
CCTGTGTGCCCTGGTTTGGTTAGAGAGCTCTAGCGGTCTCCTGTCCCACAGAATACCAGCCAGCCCCTGC
CCTACGTCGTGCCTCGGGCTGAGGGTGATTCAGAGGCAGGTGCCTGTGACAGTGGATGCAATTAGATCTA
ATGGGACGGAGGCCTTTCTCGTCGCCCTCGCTCCATGCCCACCCCCGCCTCCCTCAGGCACAGCAGGCGT
GGAGAGGATGCGCAGGAGGTAGGAGGTGGGGGACCCAGAGGGGCTTTGACGTCAGCCTGGCCTTTAAAGA
GGGCGCCTGCCTGGCGAGGGCTGTGGAGACAGAACTCGGGACCAC**Gaattc**tgcagatatccagcacagt
ggcggccgctcgaccGACCTTGTGAGGACTAGAGGAAGAATGCTCCTGGCTGTTTTGTACTGCCTGCTGT
GGAGTTTCCAGACCTCCGCTGGCCATTTCCCTAGAGCCTGTGTCTCCTCTAAGAACCTGATGGAGAAGGA
ATGCTGTCCACCGTGGAGCGGGGACAGGAGTCCCTGTGGCCAGCTTTCAGGCAGAGGTTCCTGTCAGAAT
ATCCTTCTGTCCAATGCACCACTTGGGCCTCAATTTCCCTTCACAGGGGTGGATGACCGGGAGTCGTGGC
CTTCCGTCTTTTATAATAGGACCTGCCAGTGCTCTGGCAACTTCATGGGATTCAACTGTGGAAACTGCAA
GTTTGGCTTTTGGGGACCAAACTGCACAGAGAGACGACTCTTGGTGAGAAGAAACATCTTCGATTTGAGT
GCCCCAGAGAAGGACAAATTTTTTGCCTACCTCACTTTAGCAAAGCATACCATCAGCTCAGACTATGTCA
TCCCCATAGGGACCTATGGCCAAATGAAAAATGGATCAACACCCATGTTTAACGACATCAATATTTATGA
CCTCTTTGTCTGGATGCATTATTATGTGTCAATGGATGCACTGCTTGGGGGATCTGAAATCTGGAGAGAC
ATTGATTTTGCCCATGAAGCACCAGCTTTTCTGCCTTGGCATAGACTCTTCTTGTTGCGGTGGGAACAAG
AAATCCAGAAGCTGACAGGAGATGAAAACTTCACTATTCCATATTGGGACTGGCGGGATGCAGAAAAGTG
TGACATTTGCACAGATGAGTACATGGGAGGTCAGCACCCCACAAATCCTAACTTACTCAGCCCAGCATCA
TTCTTCTCCTCTTGGCAGATTGTCTGTAGCCGATTGGAGGAGTACAACAGCCATCAGTCTTTATGCAATG
GAACGCCCGAGGGACCTTTACGGCGTAATCCTGGAAACCATGACAAATCCAGAACCCCAAGGCTCCCCTC
TTCAGCTGATGTAGAATTTTGCCTGAGTTTGACCCAATATGAATCTGGTTCCATGGATAAAGCTGCCAAT
TTCAGCTTTAGAAATACACTGGAAGGATTTGCTAGTCCACTTACTGGGATAGCGGATGCCTCTCAAAGCA
GCATGCACAATGCCTTGCACATCTATATGAATGGAACAATGTCCCAGGTACAGGGATCTGCCAACGATCC
TATCTTCCTTCTTCACCATGCATTTGTTGACAGTATTTTTGAGCAGTGGCTCCGAAGGCACCGTCCTCTT
CAAGAAGTTTATCCAGAAGCCAATGCACCCATTGGACATAACCGGGAATCCTACATGGTTCCTTTTATAC
CACTGTACAGAAATGGTGATTTCTTTATTTCATCCAAAGATCTGGGCTATGACTATAGCTATCTACAAGA
TTCAGACCCAGACTCTTTTCAAGACTACATTAAGTCCTATTTGGAACAAGCGAGTCGGATCTGGTCATGG
CTCCTTGGGGCGGCGATGGTAGGGGCCGTCCTCACTGCCCTGCTGGCAGGGCTTGTGAGCTTGCTGTGTC
GTCACAAGAGAAAGCAGCTTCCTGAAGAAAAGCAGCCACTCCTCATGGAGAAAGAGGATTACCACAGCTT
GTATCAGAGCCATTTATAAAAGGCTTAGGCAATAGAGTAGGGCCAAAAAGCCTGACCTCACTCTAACTCA
AAGTAATGTCCAGGTTCCCAGAGAATATCTGCTGGTATTTTTCTGTAAAGACCATTTGCAAAATTGTAAC
CTAATACAAAGTGTAGCCTTCTTCCAACTCAGGTAGAACACACCTGTCTTTGTCTTGCTGTTTTCACTCA
GCCCTTTTAACATTTTCCCCTAAGCCCctagagggcccgtttaaacccgctgatcagcctcgactgtgcc
ttctagttgccagccatctgttgtttgcccctcccccgtgccttccttgaccctggaaggtgccactccc
actgtcctttcctaataaaatgaggaaattgcatcgcattgtctgagtaggtgtcattctattctgggg
gtggggtggggcaggacagcaagggggaggattgggaagacaatagcaggcatgctggggatgcggtggg
ctctatggcttctgaggcggaaagaaccagctggggctctagggg**gtatcc**cccacg

Supplementary Table 1. Nomenclature of pigmented catecholaminergic cell groups and qualitative assessment of intracellular NM levels in adult tgNM mice.

Region	Name/Location	Neuromelanin levels
A1	Medullary reticular formation	+++
A2	Dorsal Vagal Complex (DVC)	+++
A6	Locus Coeruleus (LC)	+++
A8	Retrorubral field (RRF)	+++
A9	Substantia Nigra (SN)	+++
A10	Ventral Tegmental Area (VTA)	+++
A10dc	Periaqueductal gray (PAG)	++
A11	Periventricular nucleus (hypothalamus)	+
A12	Arcuate nucleus (hypothalamus)	++
A13	Zona incerta (thalamus)	+
A14-15	Preoptic periventricular nucleus	+
A16	Olfactory bulb (OB)	++

Supplementary Table 2. Primary antibodies used for immunohistochemistry (IHC), immunofluorescence (IF) and Western blot (WB).å

Primary Antibody	Manufacturer	Dilution	Research Resource Identification Portal (RRID)
**Anti-TH**	Calbiochem (657012)	1:1000 (WB)<br>1:40000 (IHC for SN-VTA and LC)<br>1:5000 (IHC for Str)<br>1:3000-1:20000 depending on animal's age (IHC for DVC)<br>1:1000 (IF)	RRID:AB_2201407
**Anti-DAT**	Chemicon #MAB369	1:500 (WB)<br>1:500 (IHC)	RRID:AB_2190413
**Anti-VMAT2**	Courtesy of G. Miller, Emory University	1:20000 (IHC)	-
**Anti-ChAT**	Millipore #AB144P	1:500 (IHC for PPN and NBM)<br>1:100-1:2500 depending on animal's age (IHC for DVC)	RRID:AB_2079751
**Anti-TPH**	Chemicon #AB1541	1:500 (IHC)	RRID:AB_90754
**Anti-GFAP**	Sigma-Aldrich #G3893	1:1000 (IHC)	RRID:AB_477010
**Anti-Iba-1**	Wako #019-19741	1:1000 (IHC)	RRID:AB_839504
**Anti-p62**	Progen #GP62-C	1:500 (IF)	RRID:AB_2687531
**Anti-Ubiquitin**	Dako #Z0458	1:500 (IF)	RRID:AB_2315524
**Anti-alpha-synuclein**	BD Biosciences #610786	1:500 (IF)	RRID:AB_398107
**Cathepsin D/ ProCathepsin D**	Sigma-Aldrich #C0715	1:1000 (WB)	RRID:AB_258707
**LC3I/LC3II**	Novus #NB100-2220	1:1000 (WB)	RRID:AB_10003146
**Anti-beta-actin**	Sigma Aldrich #A5441	1:10000 (WB)	RRID:AB_476744

Supplementary Table 3. MRM acquisition settings. RT, retention time; CV, cone voltage; CE, collision energy; CpV, capillary voltage. aParent mass after loss of water; bDetected in negative mode.

Analyte	MRM transition (m/z)	MIX	RT (min)	CV (V)	CE (eV)	CpV (kV)
**ACh**	145,98 > 86,80	ACh	1,5	10	15	3
**ACh-d4**	150 > 91	ACh	1,5	28	12	3
**NE[b]**	151,75 > 106,94	1	0,69	15	20	0,5
**DA-d4 (IS)**	157,83 > 94,8	1	1,44	10	20	0,5
**DA**	153,93 > 90,57	1	1,46	10	20	0,5
**L-DOPA**	198,1 > 152,1	1	1,48	15	15	0,5
**3MT[b]**	150,7 > 90,96	1	3,09	35	20	0,5
**AC**	149,61 > 121,91	1	3,36	25	25	0,5
**DOMA[c]**	182,86 > 136,85	2	1,62	20	14	2
**VMA[c]**	197 > 136,9	2	3,61	20	20	2
**DOPAC[c]**	166,99 > 122,82	2	3,72	18	22	2
**5SCDA**	273,1 > 166,9	3	1,73	20	20	0,5
**5SCD**	317 > 154,86	3	2,01	24	30	0,5
**5-HT**	177 > 160	4	0,97	10	5	3
**5-HT-d4 (IS)**	181 >164	4	0,97	10	5	3
**5-HIAA**	192 > 146	4	1,5	25	20	3
**Trp**	205 > 188	4	2,1	15	10	3
