# Supplementary material for: Modelling human neuronal catecholaminergic pigmentation in rodents recapitulates age-related neurodegenerative deficits
Source: Nat Commun. 2024 Oct 11;15:8819. doi: 10.1038/s41467-024-53168-7 (PMC11470033; doi:10.1038/s41467-024-53168-7)
Supplement: Supplementary file 3 — Description of Additional Supplementary Files [file 41467_2024_53168_MOESM3_ESM.pdf]

Description of Additional Supplementary Files

File Name: Supplementary Movie 1
Description: 3D-reconstruction of a clarified tgNM brain in which high levels of unstained NM (in brown) can be seen macroscopically within PD-vulnerable catecholaminergic brain regions (SNpc/VTA, LC and DVC). Source data are provided as a Source Data file.
