# Supplementary material for: Modelling human neuronal catecholaminergic pigmentation in rodents recapitulates age-related neurodegenerative deficits
Source: Nat Commun. 2024 Oct 11;15:8819. doi: 10.1038/s41467-024-53168-7 (PMC11470033; doi:10.1038/s41467-024-53168-7)
Supplement: Supplementary file 5 — Reporting Summary [file 41467_2024_53168_MOESM5_ESM.pdf]

nature portfolio

Corresponding author(s): Miquel Vila

Last updated by author(s): May 27, 2024

Reporting Summary

Nature Portfolio wishes to improve the reproducibility of the work that we publish. This form provides structure for consistency and transparency in reporting. For further information on Nature Portfolio policies, see our Editorial Policies and the Editorial Policy Checklist.

Statistics

For all statistical analyses, confirm that the following items are present in the figure legend, table legend, main text, or Methods section.

n/a	Confirmed	
☐	☒	The exact sample size (n) for each experimental group/condition, given as a discrete number and unit of measurement
☐	☒	A statement on whether measurements were taken from distinct samples or whether the same sample was measured repeatedly
☐	☒	The statistical test(s) used AND whether they are one- or two-sided<br>*Only common tests should be described solely by name; describe more complex techniques in the Methods section.*
☒	☐	A description of all covariates tested
☐	☒	A description of any assumptions or corrections, such as tests of normality and adjustment for multiple comparisons
☐	☒	A full description of the statistical parameters including central tendency (e.g. means) or other basic estimates (e.g. regression coefficient) AND variation (e.g. standard deviation) or associated estimates of uncertainty (e.g. confidence intervals)
☐	☒	For null hypothesis testing, the test statistic (e.g. F, t, r) with confidence intervals, effect sizes, degrees of freedom and P value noted<br>*Give P values as exact values whenever suitable.*
☒	☐	For Bayesian analysis, information on the choice of priors and Markov chain Monte Carlo settings
☒	☐	For hierarchical and complex designs, identification of the appropriate level for tests and full reporting of outcomes
☒	☐	Estimates of effect sizes (e.g. Cohen's d, Pearson's r), indicating how they were calculated

Our web collection on statistics for biologists contains articles on many of the points above.

Software and code

Policy information about availability of computer code

Data collection	The data from this study was collected using the following software packages (as indicated in the corresponding methods sections): ZEN 2011, Paravision 5.1, Caseviewer 3D HISTECH.
Data analysis	The data from this study was analyzed with the following software packages (as indicated in the corresponding methods sections): GraphPad v6, Image J, ZEN 2011, Stereoinvestigator v11, Fiji, SMART 3.0 Panlab, Paravision v5.1, Caseviewer 3D HISTECH

For manuscripts utilizing custom algorithms or software that are central to the research but not yet described in published literature, software must be made available to editors and reviewers. We strongly encourage code deposition in a community repository (e.g. GitHub). See the Nature Portfolio guidelines for submitting code & software for further information.

Data

Policy information about availability of data

All manuscripts must include a data availability statement. This statement should provide the following information, where applicable:

- Accession codes, unique identifiers, or web links for publicly available datasets
- A description of any restrictions on data availability
- For clinical datasets or third party data, please ensure that the statement adheres to our policy

All raw data generated in this study are provided in the Supplementary Information/Source Data file and are available in the ZENODO database under accession code 11355659 (https://doi.org/10.5281/zenodo.11355659).

Research involving human participants, their data, or biological material

Policy information about studies with human participants or human data. See also policy information about sex, gender (identity/presentation), and sexual orientation and race, ethnicity and racism.

Reporting on sex and gender	No sex-based analysis was performed because human specimens were only used in this study for qualitative illustration and comparison with the animal model.
Reporting on race, ethnicity, or other socially relevant groupings	Not applicable
Population characteristics	The age of the human specimens used in this study is provided in the Methods section: Human post-mortem brain tissue
Recruitment	Participants were recruited at the Neurological Tissue BioBank at IDIBAPS-Hospital Clinic (Barcelona, Spain) and at Biobanco en Red de la Región de Murcia (BIOBANC-MUR) (Murcia, Spain).
Ethics oversight	All procedures were conducted in accordance with guidelines established by the BPC (CPMP/ICH/135/95) and the Spanish regulation (223/2004) and approved by the Vall d'Hebron Research Institute (VHIR) Ethical Clinical Investigation Committee (PR(AG)370/2014).

Note that full information on the approval of the study protocol must also be provided in the manuscript.

Field-specific reporting

Please select the one below that is the best fit for your research. If you are not sure, read the appropriate sections before making your selection.

- [x] Life sciences
- [ ] Behavioural & social sciences
- [ ] Ecological, evolutionary & environmental sciences

For a reference copy of the document with all sections, see nature.com/documents/nr-reporting-summary-flat.pdf

Life sciences study design

All studies must disclose on these points even when the disclosure is negative.

Sample size	Methods section / Statistical Analysis. For animal studies, sample size was calculated using the sample size calculator GRanmo (https://www.datarus.eu/aplicaciones/granmo/) based on previous experience, on what would be manageable for the study, and on the anticipated variation according to previous experience from studies using related methods.
Data exclusions	Outlier samples in HPLC measurements were identified with GraphPad Prism v6 and excluded from the analysis (Methods section / Statistical Analysis). Exclusion criteria was applied in HPLC experiments because a great technical variability was detected. In all the other experiments, outlier samples were considered as part of the biological variation and consequently not excluded.
Replication	All experimental findings were reliably reproduced. Some of the reported experiments were repeated and reproducibility was confirmed. When replication was not succesful, third experiments were performed to discard changes and consequently presented as such.
Randomization	Mice were randomly distributed into the different experimental groups and control and experimental groups were processed at once to minimize bias (Methods section / Animals).
Blinding	An observer blind to the experimental group performed the quantifications as indicated in the Methods section.

Reporting for specific materials, systems and methods

We require information from authors about some types of materials, experimental systems and methods used in many studies. Here, indicate whether each material, system or method listed is relevant to your study. If you are not sure if a list item applies to your research, read the appropriate section before selecting a response.

Materials & experimental systems

n/a	Involved in the study	
☐	☒	Antibodies
☒	☐	Eukaryotic cell lines
☒	☐	Palaeontology and archaeology
☐	☒	Animals and other organisms
☒	☐	Clinical data
☒	☐	Dual use research of concern
☒	☐	Plants

Methods

n/a	Involved in the study	
☒	☐	ChIP-seq
☒	☐	Flow cytometry
☐	☒	MRI-based neuroimaging

Antibodies

Antibodies used	Antibodies catalog numbers and additional information are given in the Methods sections/Suplpementary Information Files for Immunohistochemistry, Immunofluorescence and Immunoblot.
Validation	All the antibodies used in this study have been previously reported and are commercially available. All of them have an online data sheet reporting the validity for the species and application tested.

Animals and other research organisms

Policy information about studies involving animals; ARRIVE guidelines recommended for reporting animal research, and Sex and Gender in Research

Laboratory animals	Animal species, strain, sex and age are reported in the Methods section / Animals. In each figure legend, the age of the animals at sacrifice is specified.
Wild animals	The study did not involve wild animals.
Reporting on sex	Sex was considered in the study design as a potential covariate and thus, sex was evenly distributed in the different experimental groups to avoid a potential sex-bias effect. The study design did not consider sex as an experimental variable because no sufficient number of animals for each sex was analyzed, and thus sex-based analysis are not reported.
Field-collected samples	The study did not involve samples collected from the field.
Ethics oversight	All animal experimental procedures were performed in strict accordance with protocols approved by the Vall d'Hebron Research Institute (VHIR) Ethical Experimentation Committee and the Generalitat de Catalunya (Protocol 11442), as well as the local CELYNE Ethics Research Committee of the Université Claude Bernard Lyon 1 (Protocol APAFIS#20701) for the sleep study.

Note that full information on the approval of the study protocol must also be provided in the manuscript.

Plants

Seed stocks	N/A
Novel plant genotypes	N/A
Authentication	N/A

Magnetic resonance imaging

Experimental design

Design type	Resting state
Design specifications	Acquisition time of 1h per image
Behavioral performance measures	N/A

Acquisition

Imaging type(s)	Structural
Field strength	7T
Sequence & imaging parameters	Spin-echo images from 7 continuous slices in the axial plane were acquired using the following paramteters (FOV=1.92×1.92 cm2; MTX=128×128, ST= 0.25 mm, TE=7 ms; TR= 500 ms, spatial resolution of 150×150×250 µm3) as stated in the Methods Section.
Area of acquisition	*State whether a whole brain scan was used OR define the area of acquisition, describing how the region was determined.*

Diffusion MRI ☐ Used ☒ Not used

Preprocessing

Preprocessing software	N/A
Normalization	N/A
Normalization template	N/A
Noise and artifact removal	N/A
Volume censoring	N/A

Statistical modeling & inference

Model type and settings	N/A
Effect(s) tested	N/A

Specify type of analysis: ☐ Whole brain ☒ ROI-based ☐ Both

Anatomical location(s): Substantia nigra and Locus coeruleus

Statistic type for inference (See Eklund et al. 2016)	N/A
Correction	N/A

Models & analysis

n/a	Involved in the study
☒	☐ Functional and/or effective connectivity
☒	☐ Graph analysis
☒	☐ Multivariate modeling or predictive analysis
